# Hexokinase 2 promotes ISGylation of Acyl-CoA synthetase long-chain family member 4 in sepsis-induced microglia cells

**DOI:** 10.1016/j.jlr.2025.100776

**Published:** 2025-03-12

**Authors:** Guangyang Bai, Shun Ke, Jun Lu, Shanshan Yu, Shusheng Li, Minghao Fang, Jianmin Ling

**Affiliations:** 1 Department of Emergency Medicine, Tongji Hospital, Tongji Medical College, Huazhong University of Science and Technology, Wuhan, China; 2 Department of Critical Care Medicine, Tongji Hospital, Tongji Medical College, Huazhong University of Science and Technology, Wuhan, China

**Keywords:** Hexokinase 2, Sepsis-Associated Microglial, Acyl-CoA Synthetase Long-chain Family Member 4, ISG15, ISGylation

## Abstract

Metabolic reprogramming is often observed in sepsis-associated microglial cells. However, little is known about the aberrant metabolic genes involved in neuroinflammation and lipid accumulation in microglial cells of sepsis-associated encephalopathy (SAE). Here, we show that hexokinase 2 (HK2) is upregulated and strongly associated with the inflammatory response and lipid metabolism in lipopolysaccharide-induced BV2 cells. Downregulation of HK2 lowered the activation of NOD-like receptor signaling family pyrin domain containing 3, both in BV2 cells and in the hippocampus of cecal ligation and puncture-induced male septic mice. Moreover, the inhibition of HK2 promoted lipid droplet reduction. Mechanistically, HK2 knockdown in microglial cells reduced the ISGylation of Acyl-CoA Synthetase Long-chain Family Member 4 (ACSL4) by interferon-stimulated gene 15 (ISG15). Notably, siISG15 effectively down-regulated the expression of ACSL4 in lipopolysaccharide-induced BV2 cells. Our findings provide new mechanistic insights into HK2 in microglial cells through regulation of ACSL4 ISGylation, suggesting a promising therapeutic strategy for treating SAE by targeting HK2. Our findings suggest that HK2 modulates ISGylation of ACSL4 in sepsis-induced microglial cells, indicating that therapeutic targeting of HK2 may constitute a promising strategy for SAE.

Sepsis-associated encephalopathy (SAE) is a severe neurological disorder marked by brain dysfunction due to sepsis. This condition is life-threatening and arises from the brain's abnormal response to infection (1). Patients with SAE often exhibit an acute or chronic onset of brain symptoms, ranging from cognitive impairment and delirium to coma (2). SAE involves a complicated pathophysiology with multiple mechanisms contributing to brain dysfunction and physical harm. (3). Increasing evidence indicates that patients with SAE exhibit several metabolic disease characteristics. Impaired glucose and lipid metabolism in the brain are typical features of SAE (4, 5, 6). Therefore, exploring metabolic reprogramming in SAE and discovering potential targets to enhance the quality of life and survival rates of patients with SAE is of scientific and clinical relevance.

The brain has a high energy demand in which cells form complex metabolic networks with distinct metabolic profiles (7). Microglia serve as the primary immune cells in the brain and are crucial protectors of brain function. They respond to external stimulation with varying metabolic states (8). Proinflammatory microglia rely on aerobic glycolysis to enhance the production of inflammatory cytokines, while immunoregulatory microglia boost fatty acid metabolism to facilitate anti-inflammatory actions. (9). Dysfunction of microglial metabolic activity is associated with SAE (10). Alterations in cellular metabolism are essential for influencing microglial activity during various types of stimulation or disease states. However, the precise mechanisms by which cellular metabolism controls microglial function during septicemia remain poorly understood.

Brain cells rely primarily on glucose as their primary fuel source. Hexokinase (HK) is the enzyme responsible for catalyzing the phosphorylation of glucose and acts as the rate-limiting step in glucose utilization (11). Hexokinase 2 (HK2), which is predominantly expressed in the brain, muscle, and fat tissues, has been implicated in driving glycolysis to promote inflammatory responses (12). Research demonstrates that lipopolysaccharide (LPS) rapidly upregulates HK2 expression in microglial cells, and inhibitors of HK2, such as 3-bromopyruvate (3BP) and small interfering RNA (siRNA) targeting HK2, attenuate LPS-induced microglial cell activation (13). The lack of HK2 in microglial cells helps rescue cognitive impairment in mice (7). Transcriptomic and targeted metabolomic analyses of microglial cells have suggested that suppression of HK2 is associated with activated lipid metabolism by upregulating lipoprotein lipase expression (7). As a critical metabolic checkpoint, HK2 is crucial in regulating glucose metabolism, lipid metabolism, and mitochondrial function, thereby influencing microglial cell function and maintaining brain homeostasis (9). HK2 binds to the voltage-dependent anion channel (VDAC) on the mitochondrial outer membrane, affecting the movement of metabolic products and nucleotides. (14). The activation of the NOD-like receptor family pyrin domain containing 3 (NLRP3) inflammasome is linked to the release of mitochondrial DNA (mtDNA), highlighting the intricate interplay between mitochondrial function and inflammatory responses (15). HK2 activates the Acyl-CoA Synthetase Long-chain Family Member 4 (ACSL4)-fatty acid beta-oxidation pathway by enhancing acetyl-CoA (16). HK2 is responsible for a major portion of interferon-stimulated gene 15 (ISG15)-mediated glycolytic flux (17). The ubiquitin-like protein ISG15 responds to LPS, and ISGylation is deeply involved in managing the biological processes induced by LPS. (18, 19). However, the exact function of HK2 in microglial activity during SAE is still not well comprehended. Further research is needed to elucidate the involvement of HK2 in SAE microglial function and its potential implications in the pathogenesis and treatment of SAE.

In this investigation, we detected a specific enhancement in HK2 expression within LPS-stimulated BV2 cells and the microglia of cecal ligation and puncture (CLP) mice. Genetic silencing or pharmacological inhibition of HK2 was found to be linked to the modulation of NLRP3 activation and lipid droplet aggregation in microglial cells of sepsis. Additionally, In LPS-induced BV2 cells, siHK2 lowered the levels of ACSL4 and ISG15. Moreover, siHK2 downregulated the ISGylation of ACSL4 in vitro. Consistently, siISG15 effectively downregulated the expression of ACSL4. Together, our findings demonstrate a novel role for HK2 in the ISGylation process of ACSL4 and propose a potential therapeutic approach for SAE.

## Materials and methods

### Animal and drug administration

Two-month-old male SPF C57BL/6 mice (20–22 g) were obtained from Shulaibao Biotechnology Co., Ltd. The mice were kept in an environment with a 12-h light and dark cycle, where they could freely access standard rodent food and water. The temperature was controlled between 18 and 22°C, and the humidity was kept at 50%–60%. Five mice were housed per cage. After three days of the Sham/CLP surgery, 3BP was administered intraperitoneally for seven days at a dose of 5 mg/kg or an equivalent volume of the appropriate vehicle control (20). Then, the mice were sacrificed for biochemical testing. Animal experiments were conducted according to the Guidelines for the Care and Use of Laboratory Animals of the Ministry of Science and Technology of the People's Republic of China and were approved by the Institutional Animal Care and Use Committee at Tongji Medical College, Huazhong University of Science and Technology. Patient data were excluded from the study.

### Cecal ligation and puncture

A sepsis model using CLP was established as previously explained (21). After anesthesia, hair removal, and disinfection, the cecum was isolated and punctured using a 22-gauge needle to induce septicemia. A small amount of feces was extruded, and the abdominal wall was subsequently closed. The mice were then administered 1 ml of sterile physiological saline for recovery. The sham-operated group had the same surgery but without CLP.

### Cell culture and stimulations

#### Primary neuron culture

The hippocampus of rat embryos (E17) was dissected and gently minced in Hank's Balanced Salt Solution, followed by suspension in a 0.25% (v/v) trypsin solution at 37°C for 15 min. The cells were then seeded at a density of 30,000–40,000 cells per well in 6-well plates coated with poly-D-lysine/laminin (Sigma-Aldrich). Neurobasal medium (Thermo Fisher Scientific) was used as the culture medium, with the addition of 2% B27 (Thermo Fisher Scientific), 0.5 mM glutamine, and 25 mM glutamate.

#### Primary microglia culture

Pups aged 1–3 days postnatal were utilized to create mixed glial cultures. Cells were placed in poly L-lysine-coated flasks and cultured in fresh Dulbecco's Modified Eagle Medium (DMEM, Gibco) with 10% fetal bovine serum (FBS, Gibco) added. After three days, the medium was switched to 25 ng/ml GM-CSF with 10% FBS and primary microglial cells were gathered after 12 days of culture.

#### Primary astrocytes culture

Brain tissue from C57BL/6 mice aged 1–3 days was digested, resuspended, and seeded in culture bottles. The culture medium (DMEM/F12 with 10% FBS) was changed every 3 days. After 24 h of adhesion, the cells were harvested by shaking and re-planted for further experiments.

#### BV2 culture

BV2 mouse cell lines were grown in DMEM with 10% FBS and 1% penicillin/streptomycin at 37°C in a humidified environment with 5% CO_2_.

#### 3BP and LPS treatment

3BP (376817-M, Sigma-Aldrich) and LPS (L2880, Sigma-Aldrich) were used in the cell models mentioned above. Cells were treated with 3BP (20 μm) for 30 min followed by LPS (1 μg/ml) treatment for 12 h. DMSO served as the vehicle control for the treatment conditions.

#### siRNA and LPS treatment

BV2 cells were treated with siHK2 for 24 h followed by LPS (1 μg/ml) treatment for 12 h. BV2 cells were treated with siISG15 for 24 h followed by LPS (1 μg/ml) treatment for 12 h. Negative control siRNA and DMSO served as the vehicle control for the treatment conditions.

### RNA-seg analysis

Total RNA was isolated using the TRIzol reagent (Invitrogen) following the manufacturer's protocol. RNA purity and concentration were measured with a NanoDrop 2000 spectrophotometer (Thermo Scientific), while RNA integrity was assessed using an Agilent 2,100 Bioanalyzer (Agilent Technologies). Libraries were then prepared with the VAHTS Universal V6 RNA-seq Library Prep Kit as per the 's guidelines. Transcriptome sequencing and analysis were performed by OE Biotech Co., Ltd., using the Illumina Novaseq 6,000 platform, which produced 150 bp paired-end reads. Initial raw reads in fastq format were processed using fastp, with low-quality reads filtered out to obtain clean reads. Clean reads were then aligned to the reference genome via HISAT2. Gene FPKM values were calculated, and read counts for each gene were generated using HTSeq-count.

### Immunofluorescence analysis

Mice were anesthetized using isoflurane. Following blood flushing with phosphate-buffered saline (PBS) and fixation with 4% paraformaldehyde. Then, the brains were removed and further fixed in 4% paraformaldehyde for 24 h. The brains werethen dehydrated in a 30% sucrose-PBS solution for 48 h and sectioned into 8 μm slices. Cultured cells were washed with PBS for 3 times, then fixed with 4% paraformaldehyde for 5 min. Next, the slices and cells were permeabilized and blocked in a 3%BSA solution containing 0.1%–0.5% Triton X-100 for 1 h. Then, incubated with the primary antibodies overnight at 4°C. After washing with PBS, the secondary antibody was added and incubated at room temperature for 1 h. Wash the samples again using PBS and counterstain with DAPI for 10 min. Image acquisition is performed using a Nikon SV120 microscope or a Leica 780 confocal microscope. Automated fluorescence intensity normalization was performed using image analysis software (ImageJ). Use ImageJ software to calculate the mean fluorescence intensity of each region and perform the corresponding normalization. Corresponding normalization refers to using the Sham or the Control group as a benchmark to calculate the ratio of the fluorescence intensity of the experimental samples, in order to obtain accurate quantitative results. Primary antibodies used for immunofluorescence staining include: HK2 (rabbit, 1:300, Abcam, ab209847), Iba1 (mouse, 1:500, Abcam, ab283319), GFAP (mouse, 1:500, Abcam, ab279290), Neun (mouse, 1:500, Abcam, ab104224), Plin2 (rabbit, 1:500, Zenbio, 381796), Bodipy (Beyotime, C2055), ACSL4 (rabbit, 1:200, Proteintech, 22401-1-AP), ISG15 (mouse, 1:200, Santa cruz biotechnology, sc-166712).

### Bodipy staining

Stain BV2 cells with BODIPY™ 493/503 (D2191, Thermo Fisher). In brief, BV2 cells were plated on poly-L-lysine-coated glass coverslips and treated with siHK2 for 24 h followed by LPS (1 μg/ml) treatment for 12 h. Negative control siRNA and DMSO served as the vehicle control for the treatment conditions. Dilute Bodipy with serum-free cell culture medium to obtain a 5 μM working solution. Extract the surplus culture medium from BV2 cells, introduce 100 μl of the working solution, gently shake to cover the cells completely, incubate at room temperature for 15 min, and rinse twice with culture medium for 5 min each. A confocal scanning laser microscope (VS200, Olympus) with CellSens imaging software (Olympus) was used to photograph four randomly selected visual fields per coverslip at 20x magnification. Automated fluorescence intensity normalization can be performed using image analysis software (ImageJ). Use ImageJ software to calculate the average fluorescence intensity of each region and perform the corresponding normalization. Corresponding normalization refers to using the cells of the control group as a benchmark to calculate the ratio of the fluorescence intensity of the experimental sample to that of the control group, to obtain accurate quantitative results.

### Western blotting

The lysis of tissues and cells was performed with RIPA lysis buffer (P0013B, Beyotime). After centrifugation, the protein extract was obtained from the supernatant. The proteins extracted were analyzed via SDS-PAGE. Membranes were blocked with 5% BSA, and hybridization involved primary and secondary antibodies conjugated with horseradish peroxidase. The membrane underwent three washes with PBS-T, and the immune complex was detected using enhanced chemiluminescence reagents. Primary antibodies used for immunostaining included the following: HK1 (rabbit, 1:1000, Proteintech, 15656-1-AP), HK2 (rabbit, 1:1000, Abcam, ab209847), HK3 (mouse, 1:1000, Proteintech, 67803-1-Ig), NLRP3 (rabbit, 1:1000, Abcam, ab263899), IL-1β (rabbit, 1:500, Abcam, ab283818), ACSL4 (rabbit, 1:1,000, Proteintech, 22401-1-AP), ISG15 (mouse, 1:500, santa cruz biotechnology, sc-166712), Actin (rabbit, 1:1000, Proteintech, 22401-1-AP), and GAPDH (mouse, 1:5000, Proteintech, 60004-1-Ig).

### siRNA transfection

Small interfering RNAs against HK2, ISG15, negative control siRNA, and siGAPDH were purchased from OBiO Scientific Service. These agents were transfected into BV2 cells using CALNP RNAi in vitro (D-Nano Therapeutics, DN001-05). After 24–48 h, the cells were used for further experiments. The sequences used were as follows: siHK2 sense: 5′-GCACCGAAUCUGCCAGAUUTT-3′, siISG15 sense 5′- CGGUGUCAGAACUGAAGAATT-3′, negative control siRNA (Vector) sense: 5′-UUCUCCGAACGUGUCACGUTT-3′.

### Co-Immunoprecipitation

Wash BV2 cells three times with sterile PBS to remove any residual culture medium. Add RIPA lysis buffer and incubate on ice for 10 min to ensure complete cell lysis. Centrifuge the lysate at 12,000 rpm for 10 min, then collect the supernatant and place it in a new EP tube. Quantify the protein concentration using the BCA method and adjust all samples to a uniform concentration of 1 mg/ml. Take an appropriate volume of the lysate, add non-specific IgG or antibody-free beads (agarose or magnetic beads), and incubate at 4°C for 1 h with gentle rotation. Centrifuge to remove the beads and collect the supernatant. Add the specific primary antibody to the pre-cleaned lysate, mix gently, and incubate overnight at 4°C. Add pre-treated agarose beads (pre-bound with the primary antibody) to the lysate, mix gently, and continue incubation at 4°C for 1 h. Collect the bead-bound complexes by centrifugation and wash them three times with washing buffer (RIPA + 0.1% Triton X-100) to remove any unbound proteins. Resuspend the beads in 4× SDS-PAGE sample buffer and heat at 100°C for 10 min to dissociate the proteins. Load an appropriate amount of the sample onto an SDS-PAGE gel for electrophoretic separation. Transfer the separated proteins to a nitrocellulose (NC) membrane. Block the membrane with rapid blocking solution at room temperature for 15 min. Incubate the membrane with the appropriate secondary antibody at room temperature for 1 h. Wash the NC membrane three times with TBST, 10 min each. Place the NC membrane in the ECL-WB scanner, set the exposure parameters and save the location, initiate the exposure program, and record the results. Verify the molecular weights of the protein bands.

### Statistical analysis

Specific statistical tests are used as indicated in the legend to calculate *P* values. Statistical significance was determined with a *P*-value under 0.05, and GraphPad Prism software was utilized for generating analyses and graphs.

## Results

### HK2 participates in inflammation response and lipid metabolism in LPS/CLP-induced sepsis models

**Fig. 1:**
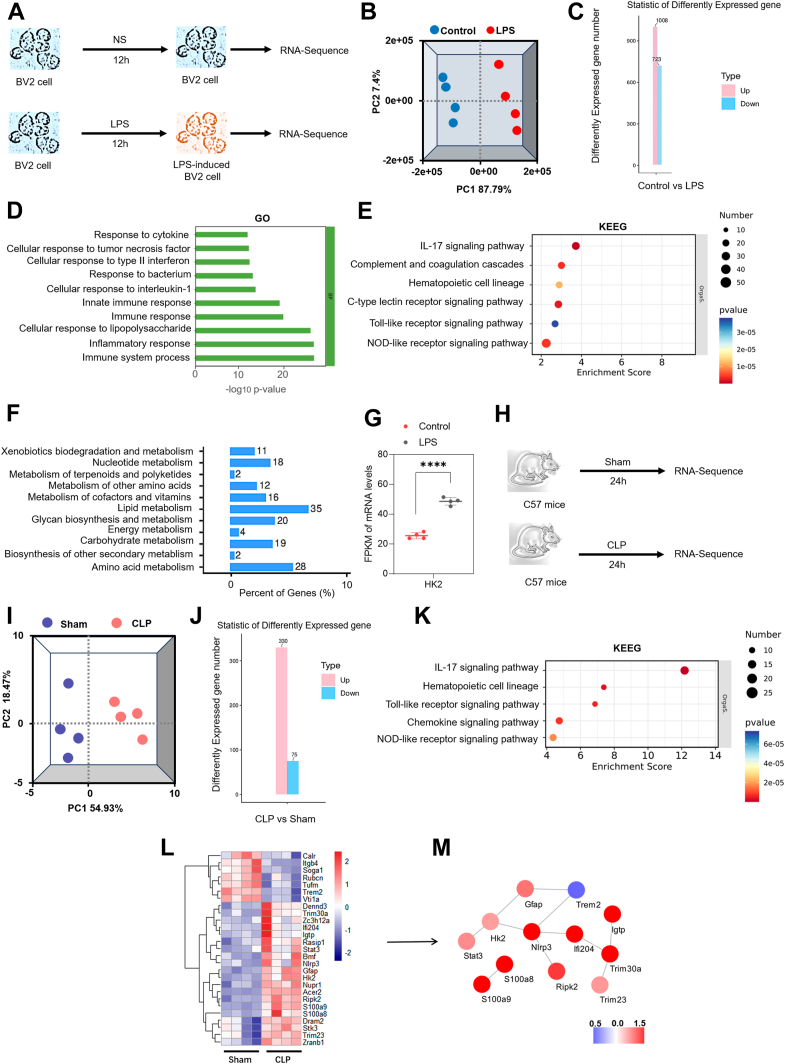
HK2 associated with inflammation and metabolism in SAE. A: BV2 cells were treated with LPS (1 μg/ml) for 12 h. DMSO served as the vehicle control. Experimental design chart of RNA-Seq in Control and LPS-induced BV2 cells. B: PCA of the RNA-seq data showed significant differences between Control and LPS. n = 4/group. C: DEGs in Control versus LPS. n = 4/group. D: Top enriched gene pathways by GO analysis of Control versus LPS. n = 4/group. E: Top enriched gene pathways by KEGG analysis of Control versus LPS. n = 4/group. F: DEGs focused on metabolism. n = 4/group. G: HK2 expression was measured in BV2 by RNA-seq. n = 4/group. H: Experimental design chart of CLP mice RNA-Seq. I: PCA of the RNA-seq data showed significant differences between Sham and CLP. n = 4 mice/group. J: DEGs in CLP versus Sham. n = 4 mice/group. K: Top enriched gene pathways by KEGG analysis of CLP versus Sham. n = 4 mice/group. L: Heatmap revealed HK2 and NLRP3 were increased in CLP compared to Sham mice. n = 4 mice/group. M: PPI analysis found HK2 regulated NLRP3. n = 4 mice/group. Mean ± SD. Two-tailed Student's t-tests. ∗∗∗∗*P* < 0.0001.

To investigate the expression of HK2 in vitro, RNA-Seq was performed to compare the transcriptomic profiles of BV2 cells under control and administration of LPS in 1 μg/ml for 12 h (Fig. 1A). Principal component analysis (PCA) confirmed the experimental grouping by showing a clear separation between the Control and LPS groups (Fig. 1B). LPS stimulation induced significant transcriptional changes, with 1731 differentially expressed genes (DEGs): 1,008 upregulated and 723 downregulated (Fig. 1C). GO analysis showed significant enrichment of inflammatory pathways in the DEGs (Fig. 1D). The KEGG indicated upregulation of the NLRs pathway and changes in lipid metabolism (Fig. 1E, F). Notably, the expression of HK2, NLRP3, and IL-1β strongly increased in LPS-induced BV2 cells (Fig. 1G and supplemental Fig. S1). To investigate the expression of HK2 in vivo, RNA-Seq was performed to compare the transcriptomic profiles of C57 mice under Sham and CLP for 24 h (Fig. 1H). A clear separation between the Sham and CLP groups was revealed by PCA, supporting the experimental grouping (Fig. 1I). CLP stimulation induced significant transcriptional changes, with 405 DEGs: 75 downregulated and 330 upregulated (Fig. 1J). KEGG analysis indicated upregulation of the NLRs pathway (Fig. 1K). The heat Map showed that the hippocampal expression of HK2 and NLRP3 was increased in CLP mice (Fig. 1L). To find the critical proteins related to inflammation, the related proteins (defined according to unpriot database) were filtered from differentially expressed proteins for protein–protein interaction (PPI) analysis. PPI analysis suggested that HK2 positively regulated NLRP3 expression, indicating HK2's role in microglial cells in SAE (Fig. 1M).

### HK2 increased in microglia of LPS/CLP-induced sepsis models

**Fig. 2:**
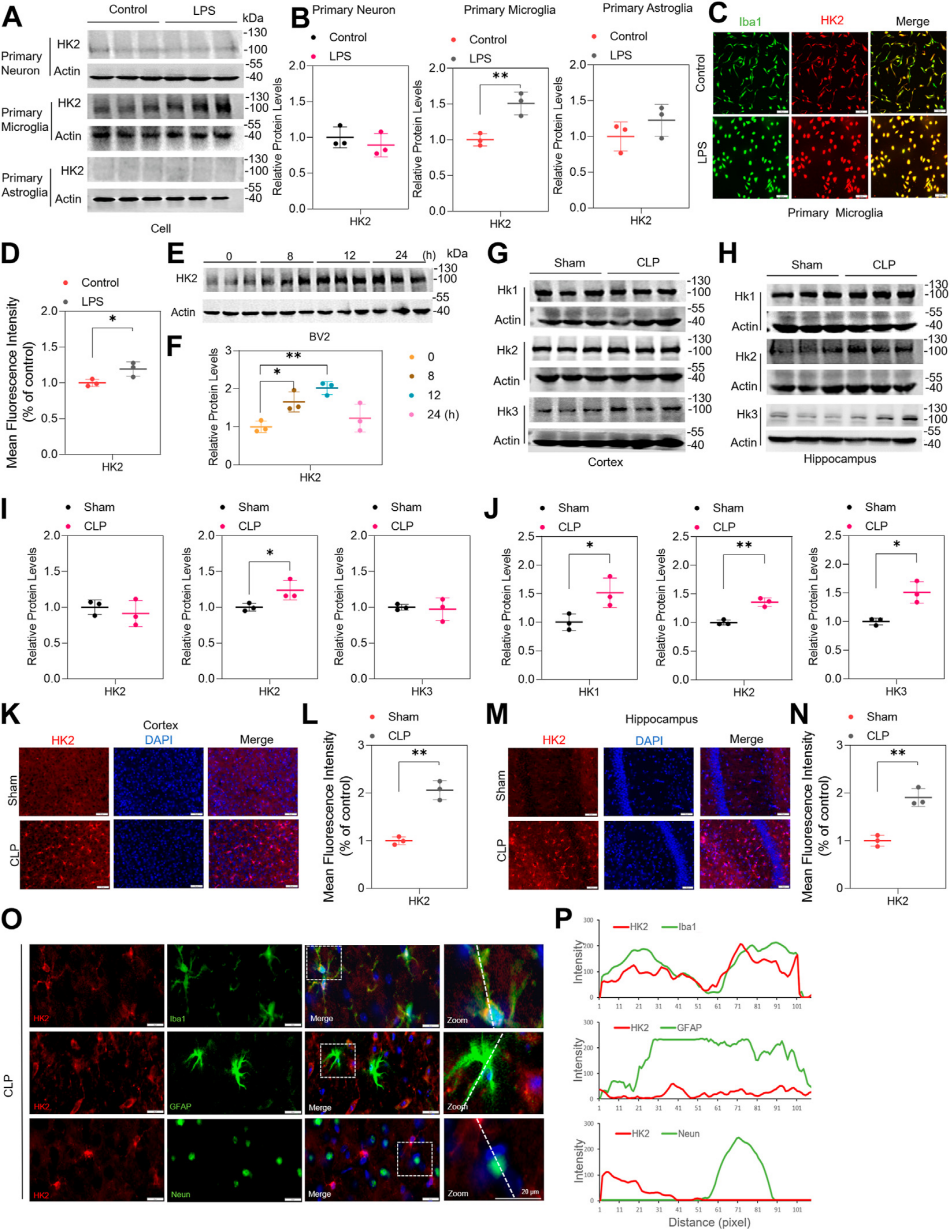
Elevated expression of HK2 in SAE. A, B: Western blot images (A) and quantifications of HK2 protein levels (B) in primary neuron, microglia, and astroglia of Control and LPS group. n = 3/group. C, D: Immunofluorescent staining images (C) and quantifications of HK2 (D) in primary microglia from Control and LPS group. n = 3/group. E and F: Time-dependent upregulation of HK2 in BV2 microglial cells exposed to LPS. Representative Western blot images (E) and quantifications of HK2 protein levels (F) in BV2 cells of Control and LPS group. n = 3/group. G, I: Western blot analysis and quantifications of HK1, HK2, and HK3 protein levels in the cortex of Sham and CLP mice. n = 3 mice/group. K, L: Immunofluorescent staining and quantifications of HK2 in the cortex from Sham and CLP mice. n = 3 mice/group. (H, J: Western blot analysis and quantifications of HK1, HK2, and HK3 protein levels in the hippocampus of Sham and CLP mice. n = 3 mice/group. (M, N) Immunofluorescent staining and quantifications of HK2 in the cortex from Sham and CLP mice. n = 3 mice/group. O, P: Immunofluorescent staining and colocalization analysis of HK2 and Iba1/GFAP/NeuN in the cortex from CLP mice. n = 3 mice/group. Mean ± SD. Two-tailed Student's *t*-tests. ∗*P* < 0.05, ∗∗*P* < 0.01.

A significant elevation in HK2 expression was detected in LPS-induced primary microglial cells, consistent with the transcriptomic data, but not in primary neurons or astroglia cells (Fig. 2A, B). Immunofluorescence assays after LPS stimulation confirmed the elevated expression of HK2 in primary microglia labeled with Iba1 (Fig. 2C, D). Additionally, the levels of HK2 were examined at different time points (0, 8, 12, and 24 h) after LPS exposure. The results showed a significant increase in HK2 levels after 8 h and 12 h of LPS exposure (Fig. 2E, F). A previous study reported the expression of HK1, HK2, and HK3 in the hippocampus and cortex of the brain (7). A significant increase in HK2 expression, but not in HK1 or HK3 expression, was observed in the cortices of CLP mice. (Fig. 2G, I). We also observed elevated HK2 protein levels in the cortex of CLP mice compared with Sham by immunofluorescence analysis (Fig. 2K, L). However, significant increases in HK1, HK2, and HK3 expressions were observed in the hippocampi of CLP mice compared to the Sham group (Fig. 2H, J). Previous studies have demonstrated that HK1 was highly expressed in neurons and astrocytes, while HK3 was expressed at low levels in the brain (22, 23). Therefore, HK1 and HK3 were not primarily expressed in microglia cells. Next, we observed elevated HK2 in the hippocampus of CLP mice compared with Sham (Fig. 2M, N). Immunofluorescence analysis additionally confirmed that HK2 was primarily present in microglia identified by Iba1, and not in astrocytes identified by GFAP or neurons identified by NeuN in the brains of CLP mice (Fig. 2O, P). The constant association of HK2 and Iba1 indicates that HK2 may be vital for the metabolic functions of microglia. In summary, these observations demonstrate that HK2 expression is upregulated in microglia due to LPS and CLP stimulation.

### Gene silence and HK2 inhibition abrogates NLRP3 activation and lipid accumulation

**Fig. 3:**
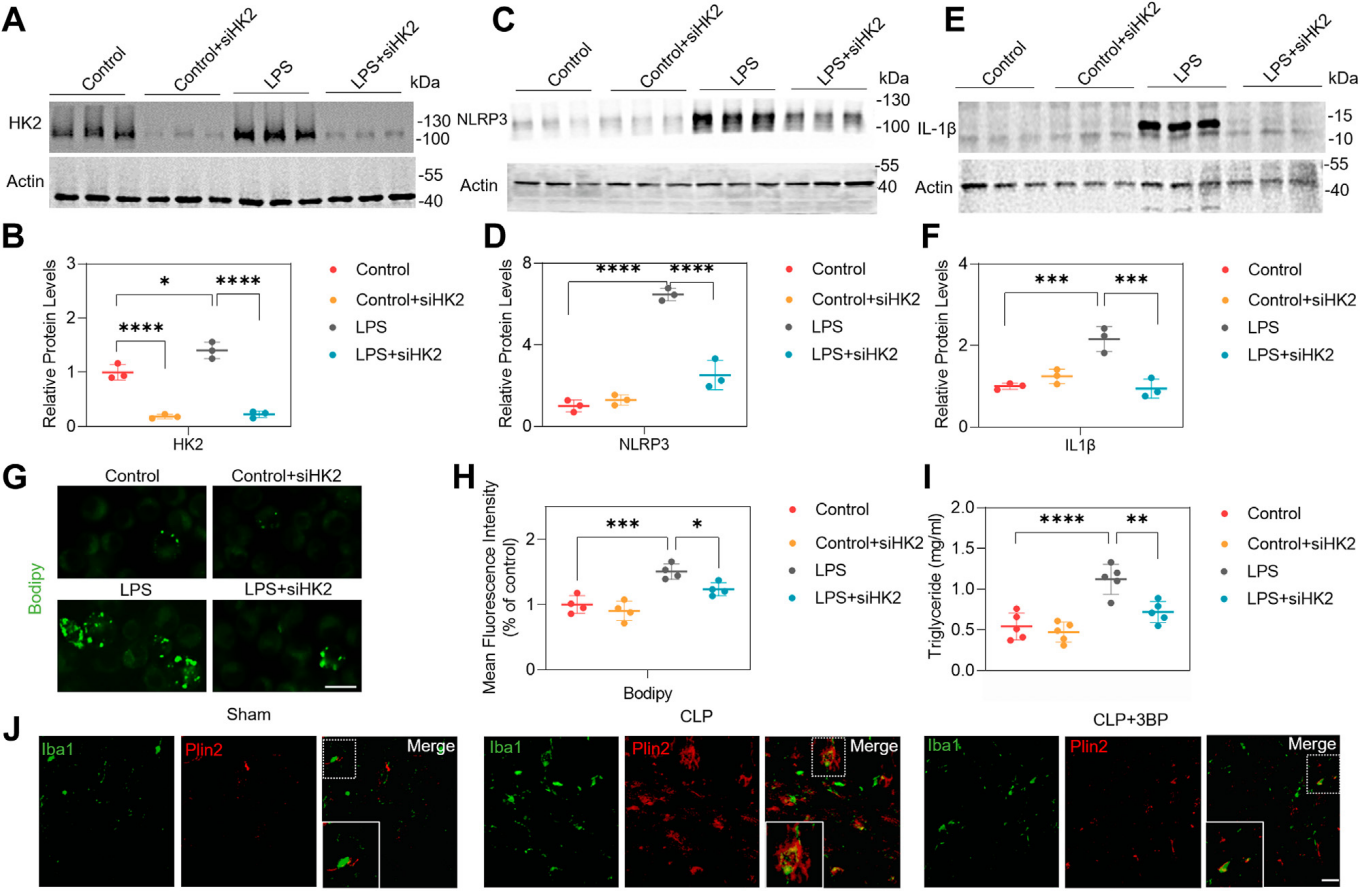
HK2 inhibition attenuates NLRP3 activation and lipid droplets accumulation. BV2 cells were treated with siHK2 for 24 h followed by LPS (1 μg/ml) treatment for 12 h. Negative control siRNA and DMSO served as the vehicle control for the treatment conditions. A, B: Representative Western blot images (A) and quantifications of HK2 protein levels (B). n = 3/group. C, D: Representative Western blot images (C) and quantifications of NLRP3 protein levels (D). n = 3/group. E, F: Representative Western blot images s (F) and quantifications of IL-1β protein levels (E). n = 3/group. G, H: Representative micrographs of Bodipy staining in BV2 cells (G) and quantification of Bodipy positive cells (H). For quantification of Bodipy-positive BV2 microglia cells, Bodipy mean fluorescence was normalized and analyzed. n = 4. Bar = 20 μm. I: Total levels of triacylglycerol (TAG) in BV2 cells cultured alone or co cultured with siHK2 for 24 h and LPS for 12 h. n = 5/group. J: Immunofluorescent staining of Iba1(green) and Plin2 (red) in hippocampi from Sham, CLP, and CLP+3BP group. n = 3. Bar = 20 μm. All data are shown as mean ± SD or SEM. One-way ANOVA followed by Tukey's post hoc test. ∗*P* < 0.05, ∗∗*P* < 0.01, ∗∗∗*P* < 0.001, ∗∗∗∗*P* < 0.0001.

To assess whether siHK2 could abrogate NLRP3 activation, BV2 cells were treated with siHK2 for 24 h followed by LPS (1 μg/ml) treatment for 12 h. Negative control siRNA and DMSO served as the vehicle control for the treatment conditions. We examined the expression of HK2 after siHK2 treatment and observed a significant reduction between the LPS and LPS+siHK2 groups (Fig. 3A, B). Additionally, a significant decrease in NLRP3 was observed in the LPS+siHK2 group than in the LPS group (Fig. 3C, D). Considering NLRP3's association with mediating IL-1β production, we sought to elucidate whether HK2 inhibition attenuated IL-1β levels. As shown in Fig. 3E, F, the expression of IL-1β was significantly lower in LPS+siHK2 cells than in LPS cells. To investigate the role of microglial HK2 in CLP-induced SAE mice, we used a specific HK2 inhibitor, 3BP, and evaluated its effect on NLRP3 expression in the brain. An intraperitoneal injection of 3BP was administered as illustrated in supplemental Fig. S2. Western blotting results further confirmed that the expression of NLRP3 decreased in the hippocampi of CLP+3BP mice than in those of CLP mice (supplemental Fig. S3A, B). In summary, these findings indicated that gene silence and HK2 inhibition abrogate NLRP3 and IL-1β activation in SAE models.

HK2 participates in lipid metabolism (7). Lipid droplet buildup in microglia is increasingly recognized as a marker of inflammation (24, 25). Bodipy is commonly used to detect lipid droplets which consist of neutral lipids. By immunofluorescence assay, we observed that LPS increased Bodipy-positive BV2 cells. SiHK2 reduced Bodipy-positive lipid droplet accumulation in LPS-induced BV2 cells (Fig. 3G, H). By triacylglycerol (TAG) assay, we observed that LPS increased total levels of TAG in BV2 cells compared to the control group, but siHK2 reduced the storage of TAG in LPS-induced BV2 cells (Fig. 3I). A previous study reported that Plin2 is a ubiquitously expressed protein that regulates lipid droplet metabolism (26). Using an immunofluorescence assay, we found that CLP increased Plin2 expression in the brain (Fig. 3J). Pharmacological inhibition of HK2 by 3BP led to reduced Plin2 expression in CLP-induced mice (Fig. 3J). Overall, these results demonstrated that gene silence HK2 in LPS-induced BV2 cells and HK2 inhibition in CLP mice are involved in lipid droplet accumulation.

### HK2 regulates ACSL4 ISGylation in LPS-treated BV2 cells

**Fig. 4:**
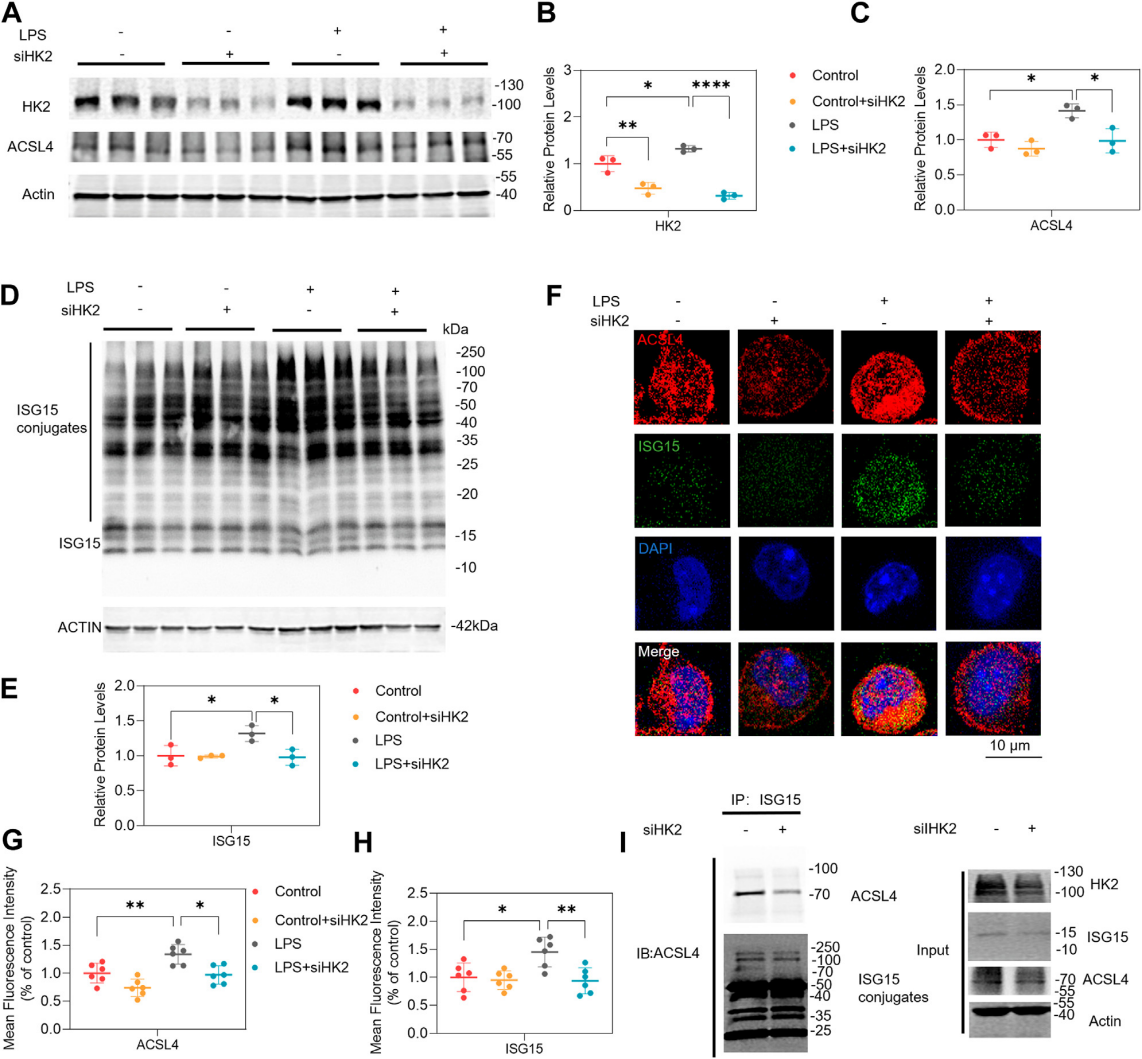
HK2 regulated ACSL4 by ISG15 and ISGylation in LPS-induced BV2 cells. BV2 cells were treated with siHK2 for 24 h followed by LPS (1 μg/ml) treatment for 12 h. Negative control siRNA and DMSO served as the vehicle control for the treatment conditions. A–C: Western blot images (A) and quantifications of HK2 (B) and ACSL4. C: protein levels in BV2 cells. n = 3/group. D and E: Western blot analysis and quantifications of ISG15 protein levels after siHK2 in vitro. n = 3/group. F–H: Representative images (F) and quantifications of ACSL4 (G) and ISG15 (H) mean fluorescence. n = 6/group. I: Co-Immunoprecipitation of ACSL4 and ISG15 protein levels after siHK2 in LPS induced BV2 cells. n = 3/group. All data are shown as mean ± SD. One-way ANOVA followed by Tukey's post hoc test. ∗*P* < 0.05, ∗∗*P* < 0.01, ∗∗∗∗*P* < 0.0001.

RNA-seq analyses indicated that the DEGs, both upregulated and downregulated, were significantly enriched in fatty acid omega-oxidation and degradation, suggesting that HK2 is involved in lipid metabolism (Fig. 1). Reactive oxygen species (ROS) influence fatty acid biosynthesis pathways to regulate lipid metabolism. Using MitoSox to assess mitochondrial ROS, it was found that ROS production was significantly higher in the LPS group than in the control, but siHK2 markedly lowered ROS levels in LPS-exposed BV2 cells (supplemental Fig. S4A, B). In addition, LPS stimulation led to decreases in superoxide dismutase (SOD) levels and siHK2 increase SOD levels in LPS-treated BV2 cells (supplemental Fig. S4C). A previous study reported that ACSL4 were expression increased in LPS-induced BV2 cells (27). Consistent with the transcriptomic data, a significant increase in ACSL4 expression was observed in the hippocampi of CLP mice (supplemental Fig. S5A, B) and LPS-treated BV2 cells (Fig. 4A, C). Western blotting revealed that siHK2 decreased ACSL4 expression in vitro (Fig. 4A–C). RNA-seq analyses revealed no significant decrease in mRNA levels of ACSL4 in LPS-induced BV2 microglial cells after HK2 inhibition (supplemental Fig. S5C). A previous study reported that ISG15 and ISGylation participate in HK2-mediated glycolysis (17). Further validation by western blotting confirmed that the protein levels of ISG15 and ISGylation increased after LPS treatment of BV2 microglial cells (Fig. 4D, E). Moreover, ISG15 and ISGylation expressions were observed to decrease in LPS-stimulated BV2 cells after siHK2 (Fig. 4D, E). Consistent with the western blotting results, the mean fluorescence of ISG15 and ACSL4 were upregulated and showed stronger colocalization in LPS-induced BV2 microglial cells compared to the control group. As expected, we found that siHK2 treatment led to a decrease in the mean fluorescence of ISG15 and ACSL4 in the LPS group (Fig. 4F–H). Additionally, we confirmed the ISG15–ACSL4 interaction using co-immunoprecipitation (Co-IP) assays. The result showed that ISG15 was combined with ACSL4. Similarly, siHK2 decreased ACSL4 ISGylation (Fig. 4I). These findings indicated the specific regulation of HK2 in ISGylation of ACSL4 in microglia cells.

### siISG15 decreased ISGylation of ACSL4 in LPS-induced BV2 cells

**Fig. 5:**
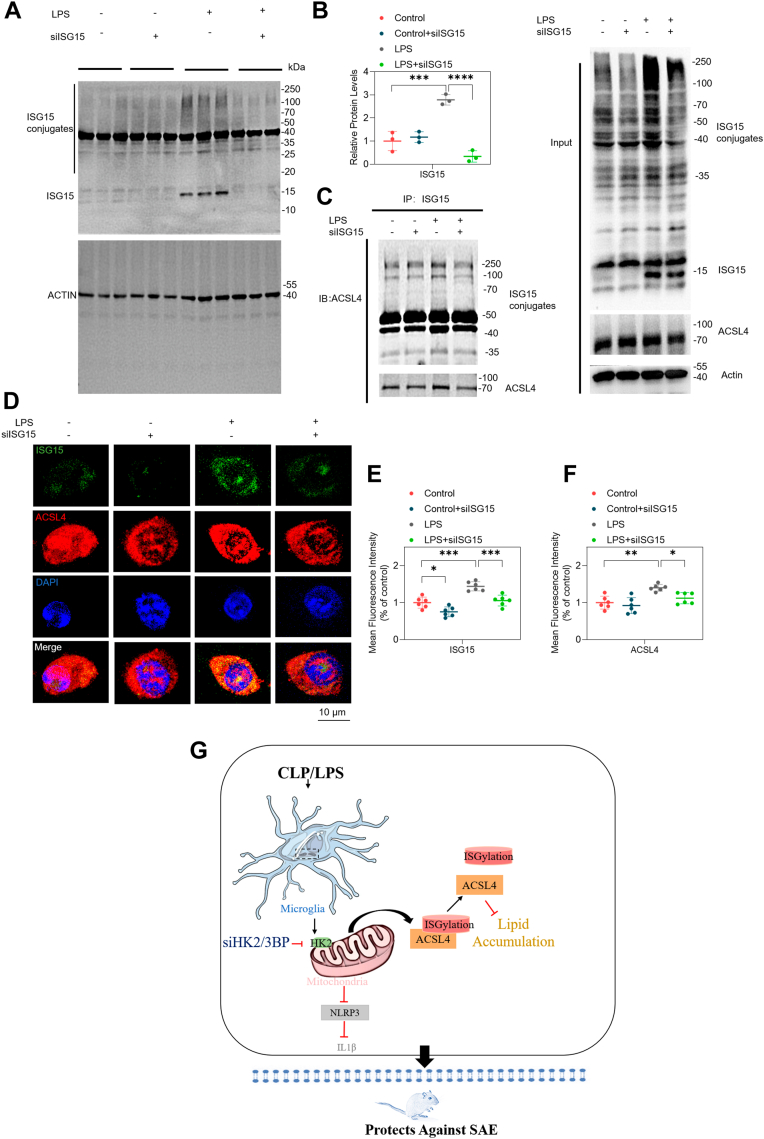
siISG15 decreased ACSL4 by alleviating ISGylated ACSL4 in LPS-induced BV2 cells. BV2 cells were treated with siISG15 for 24 h followed by LPS (1 μg/ml) treatment for 12 h. Negative control siRNA and DMSO served as the vehicle control for the treatment conditions. A, B: Western blot analysis (A) and quantifications (B) of ISG15 protein levels after siISG15 in vitro. n = 3/group. C: Co-IP of ACSL4 and ISG15 protein levels after siISG15. n = 3/group. (D–F) Representative images (D) and quantifications of normalized mean fluorescence intensity of ISG15 (E) and ACSL4 (F) in LPS-induced BV2 microglia cells after siISG15. n = 6/group. G: A schematic model of the HK2 role involved in CLP or LPS induced microglia cells. Mean ± SD. One-way ANOVA followed by Tukey's post hoc test for. ∗*P* < 0.05, ∗∗*P* < 0.01, ∗∗∗*P* < 0.001, ∗∗∗∗*P* < 0.0001.

The effect of ISG15 on ACSL4 was further examined in BV2 cells with siISG15, a siRNA containing a siISG15 sequence that could downregulate 70% of ISG15 mRNA (data not shown). We further validated the efficiency of siISG15 transfection using Western blotting in LPS-induced BV2 cells and observed that downregulates ISG15 at the protein level (Fig. 5A, B). Using Co-IP assays, we found that siISG15 succeeded in downregulating ACSL4 expression in LPS-induced BV2 cells (Fig. 5C). Furthermore, immunofluorescence assay showed that siISG15 led to a decrease in ISG15 and ACSL4 (Fig. 5D–F). What's more, ACSL4 decreased only in the presence of LPS with siISG15 treatment. These findings suggest that the specific downregulation of ISG15 could decrease the expression of ACSL4 by alleviating the ISGylation of ACSL4 in LPS-induced BV2 cells.

## Discussion

Sepsis is marked by a syndrome of systemic inflammatory response (28). During this pathological process, the release of inflammatory mediators is associated with metabolic dysfunction, which affects glucose metabolism in the brain (4). Glucose metabolism is upregulated during sepsis (29). However, there is still much to learn about how microglial inflammatory activation and metabolic reprogramming are controlled during SAE pathogenesis. In this study, we found that the expression of HK2 increased in LPS-induced primary microglial cells, BV2 cells, and the brains of CLP mice. Gene silencing of HK2 in LPS-induced BV2 cells or pharmacological inhibition of HK2 in CLP-induced mice attenuates the activation of NLRP3 and decreases ACSL4-mediated lipid droplet aggregation. Mechanistically, HK2 knockdown reduced the ISGylation of ACSL4 by decreasing ISG15 expression. Moreover, siISG15 effectively decreased the expression of ACSL4 by alleviating ISGylation of ACSL4 in LPS-induced microglial cells (Fig. 5G).

Microglia function as the brain's frontline defense, capable of reacting to environmental stimuli and exhibiting multiple functional states, requiring a rapid and adaptable energy supply (30). Hexokinases are the first rate-limiting step in glucose metabolism and exhibit different distributions in various brain cell types (11). The five isoforms of mammalian hexokinases include HK1, HK2, HK3, HK4, and a protein containing a hexokinase domain called protein-1 (31). Microglia exhibited the greatest capacity to boost brain glucose metabolism by selecting HK2 (32). Microglia take in and process more glucose compared to astrocytes and neurons (33). HK2 serves as a specific glycolytic gatekeeper in microglia. Prior research indicates that decreasing HK2 expression impairs the standard dynamics and injury-induced chemotaxis of microglia (9). Under pathological conditions, such as the proliferation of microglia after ischemia or the repopulation of activated microglia after acute depletion, the demand for ATP increases, and knockdown of HK2 inhibits the proliferation and repopulation of microglia (9). Hence, HK2 in microglia possesses the potential for swift proliferation and replenishment. (34).

The NLRP3 inflammasome serves as a fundamental signaling complex crucial for protection against infectious and sterile inflammation (35). The NLRP3 inflammasome activation is a tightly controlled process that regulates the release of the powerful pro-inflammatory cytokine IL-1β in sepsis (36). Although the NLRP3 inflammasome responds to several stimuli, the upstream signals are not easily identifiable (37). The protein HK2 has been thoroughly investigated for its ability to prevent apoptosis and stabilize mitochondria. It is usually found in excess in tumor cells and is believed to protect them from cell death (38). HK2 is a metabolic enzyme that acts as a link between various stimuli and inflammatory signaling, participating in a series of events on the mitochondrial surface that promote the activation of NLRP3 inflammasomes (39). In microglial cells, the expression of HK2 is essential for aerobic respiration based on glucose metabolism (31). HK2 is located on the outer mitochondrial membrane, where it binds to VDAC, thereby maximizing the reaction catalyzed by HK2, which uses the ATP output by VDAC for glucose phosphorylation (40). Hexokinase dissociates from mitochondria to promote the oligomerization of VDAC, facilitating the assembly and activation of NLRP3 inflammasomes. The release of HK2 from the mitochondria triggers the activation of calcium-sensitive receptors, resulting in the release of calcium from the endoplasmic reticulum and its absorption by the mitochondria. The flow of calcium into the mitochondria leads to the oligomerization of VDAC, which forms large molecular-sized pores in the outer mitochondrial membrane, leading to changes in MMP (41), allowing mtDNA, which is usually associated with inflammation, to exit the mitochondria (39). LPS stimulation results in an increase in mtDNA fragments, and changes in mitochondrial enzymes affect mtDNA production and NLRP3 inflammasome activation (15). Mitochondrial ROS plays a crucial role in intracellular signaling (42). In BV2 microglial cells, LPS stimulation may upregulate mitochondrial HK2, leading to the production of large amounts of ROS. These ROS, in turn, influence fatty acid biosynthesis pathways to regulate lipid metabolism (43, 44). HK2 may modulate lipid metabolism by regulating ROS levels, thereby affecting microglial activation and the inflammatory response. Our study found that the increase in HK2 levels in SAE was related to the activation of NLRP3. Reducing HK2 expression can alleviate the activation of NLRP3.

Under normal conditions, fatty acid oxidation accounts for approximately 20% of total energy production in the brain (45). Previous studies using transcriptomics and metabolomics have shown that the inhibition of HK2 stimulates fatty acid metabolism in microglia (7). Bodipy consists of neutral lipids. By immunofluorescence assay, we found that siHK2 reduced Bodipy-positive lipid droplets in LPS-induced BV2 cells. TheTAG assay showed that siHK2 decreased storage of TAG in LPS-induced BV2 cells. Plin2 plays a crucial role in maintaining the stability of lipid droplets and inhibiting their digestion by fatty acid enzymes. Downregulation of HK2 inhibits Plin2 and suppresses fatty acid oxidation-induced inflammation. ACSL4 plays a crucial role in regulating fatty acid metabolism by converting long-chain fatty acids into fatty acyl-CoA esters (46). ACSL4 acts on arachidonic acid which is an essential component of cellular membranes and lipid droplets (47). Some studies indicate that ACSL4 promotes intracellular lipogenesis, lipid droplets accumulation, and microglia-mediated neuroinflammation by regulating lipid metabolism (27, 48, 49). The production of proinflammatory cytokines in microglia was inhibited by knocking down ACSL4 (50). The metabolic reprogramming mediated by ACSL4 has attracted widespread attention in metabolic disease research (47). Moreover, HK2 boosts the buildup of acetyl-CoA and triggers the transcription of ACSL4, which is involved in fatty acid β-oxidation (16). In this study, we found that ACSL4 levels significantly increased in both CLP-induced brain tissue and LPS-stimulated BV2 cells. siHK2 effectively reduces the levels of ACSL4 in microglia. ISG15 is an interferon-stimulated gene induced by type I interferons and encodes a multifunctional protein that acts both as a soluble molecule and protein modifier (18). ISG15 functions as a cytokine and covalently binds to substrate proteins through a series of enzymatic reactions known as ISGylation. Non-targeted mass spectrometry studies have shown that over 300 potential substrates undergo ISGylation (51). ISG15 is also involved in signaling pathways such as HK2, NF-κB, JNK, and IRF-3 (17, 52). We found that ACSL4 underwent ISGylation, which was regulated by HK2.

This study has several limitations. First, our cell model used LPS-treated BV2 cells. The significant concern was its oncogenic transformation. BV2 cells were derived from immortalized murine microglia which may alter their phenotypic and functional properties compared to primary microglia. The oncogenic background of BV2 cells may influence their proliferation rates and energy metabolism, which could confound studies focusing on microglial activation or metabolic regulation. Second, 3BP was not a fully specific inhibitor of HK2. Third. Both HK1 and HK3 expression in the hippocampi of CLP-induced mice were upregulated. Their precise roles in SAE metabolism were still unclear.

In summary, we have demonstrated that HK2 plays a key role in the pathogenesis of LPS/CLP induce micrglias by acting as a bridge between NLRP3 activation and changes in ACSL4-mediated lipid droplet aggregation. Both ACSL4 and ISG15 levels decreased when HK2 was inhibited. Additionally, the knockdown of ISG15 in microglial cells effectively regulated the ISGylation of ACSL4. According to our data, HK2 targeting may offer a novel strategy for SAE treatment.

## Data availability

The raw sequence data have been submitted to the NCBI Gene Expression Omnibus (GEO) with accession number GSE280721. The other Data are available from the corresponding authors on reasonable request.

## Supplemental data

This article contains supplemental data.

## Conflicts of interest

The authors declare that they have no conflicts of interest with the contents of this article.
