# Supplementary material for: Hexokinase 2 promotes ISGylation of Acyl-CoA synthetase long-chain family member 4 in sepsis-induced microglia cells
Source: J Lipid Res. 2025 Mar 12;66(4):100776. doi: 10.1016/j.jlr.2025.100776 (PMC12018552; doi:10.1016/j.jlr.2025.100776)
Supplement: Supplementary material [file mmc1.pdf]

Hexokinase 2 Promotes ISGylation of ACSL4 in Sepsis-induced Microglia Cells

Guangyang Bai, Shun Ke, Jun Lu, Shanshan Yu, Shusheng Li, Minghao Fang, Jianmin Ling

Supplementary Figure 1

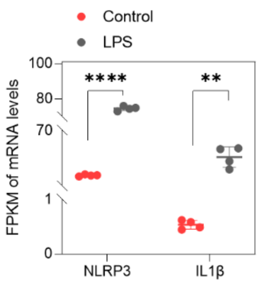

Figure S1. Inflammation genes expressions in LPS-induced BV2 cells.

NLRP3 and IL-1β expressions were measured in control and LPS groups of BV2 cell by RNA-seq. n=4 /group. Mean ± SD. Two-tailed Student's t-tests. **, $P < 0.01$, ****, $P < 0.0001$.

Supplementary Figure 2

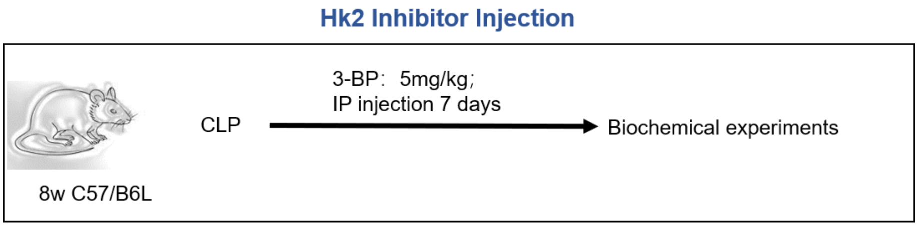

Figure S2 Schematic diagram of 3BP administration in vivo.

After three days of the Sham/CLP surgery, 3BP was administered intraperitoneally for seven days at a dose of 5 mg/kg or an equivalent volume of the appropriate vehicle control.

Supplementary Figure 3

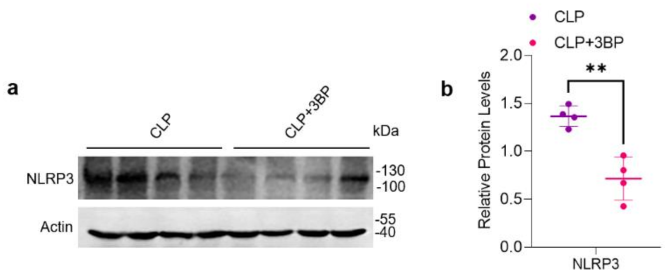

Figure S3. NLRP3 protein levels after 3BP treatment from the hippocampi of CLP and CLP+3BP mice.

(a, b) Western blot analysis and quantifications of NLRP3 protein levels after 3BP treatment from the hippocampi of CLP and CLP+3BP mice. n=4 mice /group. Data are shown as mean ± SD. Two-tailed Student's t-tests . **P < 0.01.

Supplementary Figure 4

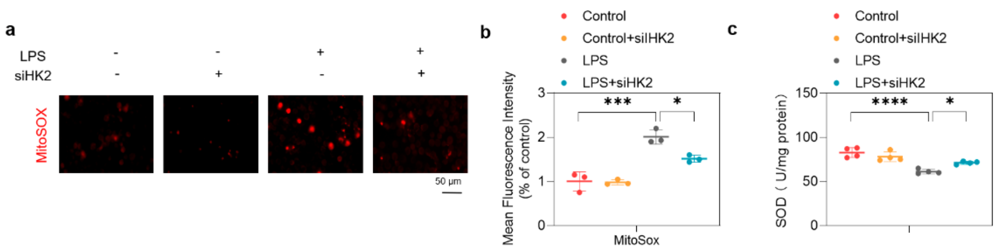

Figure S4. siHK2 attenuates oxidative stress in LPS-induced BV2 cells

BV2 cells were treated with siHK2 for 24 h followed by LPS (1 μg/mL) treatment for 12 h. Negative control siRNA and DMSO served as the vehicle control for the treatment conditions. Typical images (a) and analysis of relative fluorescence intensity of Mitosox (b), n= 3 /group. (c) Levels of superoxide dismutase (SOD) in BV2 cells, n= 4 /group. Mean ± SD. One-way ANOVA followed by Tukey's post hoc test. *P < 0.05, ***, P < 0.001, ****, P < 0.0001.

Supplementary Figure 5

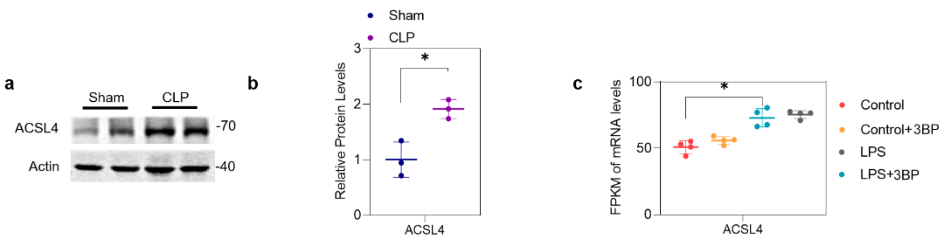

Figure S5. The ACSL4 levels in vivo and in vitro.

(a, b) Western blot analysis and quantifications of ACSL4 protein levels from the hippocampi of Sham and CLP mice, n=3 /group. (c) RNA-seq analyses revealed no significant decrease in FPKM mRNA levels of ACSL4 in LPS-induced BV2 microglial cells after HK2 inhibition by 3BP, n=4 /group. Mean ± SD. One-way ANOVA followed by Tukey's post hoc test. *P < 0.05.
